# A computational method for large-scale identification of esophageal cancer-related genes

**DOI:** 10.3389/fonc.2022.982641

**Published:** 2022-08-16

**Authors:** Xin He, Wei-Song Li, Zhen-Gang Qiu, Lei Zhang, He-Ming Long, Gui-Sheng Zhang, Yang-Wen Huang, Yun-mei Zhan, Fan Meng

**Affiliations:** ^1^ Department of Respiratory and Critical Care, The First Affiliated Hospital of Gannan Medical University, Ganzhou, China; ^2^ Department of pathology, The First Affiliated Hospital of Gannan Medical University, Ganzhou, China; ^3^ Department of Oncology, The First Affiliated Hospital of Gannan Medical University, Ganzhou, China; ^4^ Department of Gastroenterology, The First Affiliated Hospital of Gannan Medical University, Ganzhou, China; ^5^ School of Basic Medicine, Gannan Medical University, Ganzhou, China

**Keywords:** esophageal cancer, gene, graph convolutional network, logical matrix factorization, gene interaction

## Abstract

The incidence of esophageal cancer has obvious genetic susceptibility. Identifying esophageal cancer-related genes plays a huge role in the prevention and treatment of esophageal cancer. Through various sequencing methods, researchers have found only a small number of genes associated with esophageal cancer. In order to improve the efficiency of esophageal cancer genetic susceptibility research, this paper proposes a method for large-scale identification of esophageal cancer-related genes by computational methods. In order to improve the efficiency of esophageal cancer genetic susceptibility research, this paper proposes a method for large-scale identification of esophageal cancer-related genes by computational methods. This method fuses graph convolutional network and logical matrix factorization to effectively identify esophageal cancer-related genes through the association between genes. We call this method GCNLMF which achieved AUC as 0.927 and AUPR as 0.86. Compared with other five methods, GCNLMF performed best. We conducted a case study of the top three predicted genes. Although the association of these three genes with esophageal cancer has not been reported in the database, studies by other reseachers have shown that these three genes are significantly associated with esophageal cancer, which illustrates the accuracy of the prediction results of GCNLMF.

## Introduction

Esophageal cancer is a common gastrointestinal malignancy, and its common clinical symptoms include retrosternal pain and progressive dysphagia (1). Judging from its prevalence, the incidence of esophageal cancer in China is relatively high globally. The pathological type of esophageal squamous cell carcinoma is more common. The typical symptoms of esophageal cancer patients are not obvious in the early stage, and the disease progresses slowly, so it is difficult to detect early. However, when esophageal cancer develops to the middle and advanced stage, the treatment difficulty increases and the prognosis is poor (2). At present, the treatment of patients with esophageal cancer is mainly surgery, radiotherapy, and chemotherapy. The patients with advanced stage have poor curative effect and high mortality (3).

The occurrence of esophageal squamous cell carcinoma usually goes through a long-term and multi-stage development process. In the original efficient and orderly epithelial renewal cycle, carcinogenic factors are continuously exposed. The basal cells first show morphological changes, atypical hyperplasia and invasion to the surface. The squamous epithelial cells show nuclear atypia and abnormal differentiation. In the early stage of carcinogenesis, this pathological change is limited to the inner part of the mucosal layer and does not break through the basement membrane to infiltrate and invade downward. It is called squamous epithelial dysplasia and is the only recognized form of precancerous lesions of esophageal squamous cell carcinoma (4). A 13 year prospective cohort study (5) conducted a long-term follow-up of normal and precancerous people in Linzhou, Henan Province. It was found that compared with normal people, the relative risk of esophageal squamous cell carcinoma in patients with precancerous lesions (regardless of the degree of specific lesions) was 12.7 (5.5-29.6) times higher than that in normal people. Moreover, the cumulative incidence rate of esophageal squamous cell carcinoma in patients initially diagnosed with precancerous lesions at the end of the study was 58%, which was 8% in the population initially diagnosed with no abnormality. Therefore, atypical hyperplasia of squamous epithelium is a high-risk factor and predictor of esophageal squamous cell carcinoma. Timely early diagnosis of patients with precancerous lesions is an important means to reduce the incidence rate of esophageal squamous cell carcinoma. At present, regular gastroscopy screening for high-risk groups is an effective method for early diagnosis of esophageal cancer. However, due to the heterogeneity between patients, different patients with the same diagnosis still have different outcomes and outcomes. Therefore, an in-depth understanding of the causes of esophageal epithelial progression from normal to precancerous lesions to tumors and a comprehensive analysis of the molecular mechanism of tumor occurrence are of indispensable value for us to evaluate the risk of progression of patients with precancerous lesions, improve the diagnosis and cure rate of patients, and increase the means and opportunities for early diagnosis and treatment.

With the development and progress of next-generation sequencing technology, multi-omics research on tumors has become an indispensable means to explore the mechanism of tumor occurrence and development. In recent years, a number of esophageal cancer genomic studies, including the Cancer Genome Atlas (TCGA) project, have identified a large number of genomic variants in esophageal squamous cell carcinoma by performing whole-exome or whole-genome sequencing of clinically collected tumor tissue samples (6). Although these studies reveal the important role of the identified genomic alterations in ESCC, the question of how normal epithelial cells are transformed into invasive carcinomas through mutations in precancerous lesions remains unanswered due to the cross-sectional design of previous studies. Compared with studies on esophageal squamous cell carcinoma, there are still few studies on esophageal precancerous lesions. Some researchers used microdissection experimental technology to collect tumor lesions and precancerous lesions adjacent to the tumor on paraffin sections of 45 cases of esophageal squamous cell carcinoma, as well as lesions on paraffin sections of 13 precancerous lesions for full penetrance. Subgroup sequencing analysis showed that epithelial cells in the precancerous stage already have mutations similar to those of tumors, including high-frequency mutations in esophageal cancer driver genes such as TP53, NFE2L2, NOTCHI, FAT1, indicating that the precancerous stage Epithelial cells have undergone the effects of genomic variation (7). Coincidentally, in another report, the researchers performed whole-exome sequencing on 227 different pathological stages of 70 patients with esophageal squamous cell carcinoma, and also found that dysplasia and esophageal squamous cell carcinoma have similar driver genes. Moreover, they also found that there were no genomic alterations of the same type of cancer foci in the tissues of simple non-dysplasia, indicating that most of the genomic events related to canceration started from the stage of precancerous lesions (8). Researchers have performed genomic mutation studies on pathologically normal esophagus (9) and their results have shown that although the exon mutation burden of normal esophageal epithelial cells (derived from human individuals without esophageal squamous cell carcinoma) increases with age, but no cancer-related morphological changes occurred from a histopathological point of view. The results showed that although the exon mutation load of normal esophageal epithelial cells (derived from human individuals without esophageal squamous cell carcinoma) increased with age, there were no cancer-related morphological changes from the perspective of histopathology. The above studies suggest that in the overall organizational environment, the genomic changes of epithelial cells are not enough to fully explain the occurrence of esophageal cancer. Other factors such as immunosuppression in the microenvironment (TME) and cell-cell interaction may also play an important role in the occurrence of esophageal squamous cell carcinoma. A number of experimental studies and clinical analyses have also revealed the impact of TME on tumorigenesis and development in esophageal squamous cell carcinoma. Kashima et al. (10)found that the positive intensity of cancer associated fibroblasts (CAFs) was significantly positively correlated with lymph node metastasis by staining FFPE tissue sections of patients with esophageal squamous cell carcinoma, so they verified this hypothesis through in vitro experiments and in situ metastasis mouse models, CAFs can promote the metastatic ability of cancer cells and can be used as a marker of patient prognosis. Another experimental study on the microenvironment cells of esophageal squamous cell carcinoma found that the up regulation of transcription factor F0X01 can promote the polarization of macrophages from M0 to M2 by regulating the expression of CCL20 and csf1, while M2 cells play the regulatory functions of anti-inflammatory and immunosuppression, and promote the occurrence of tumors (11). Similarly, Yang et al. found that blocking the recruitment of tumor associated macrophages (TAMs) can significantly reduce the incidence of tumors in the mouse tumorigenesis model and enhance the anti-tumor effect of CD8 + T cells in the tumor microenvironment. More importantly, M2 polarization increases the expression of PD-L2 in TAMs, leading to immune evasion and tumor promotion through PD-1 signaling pathway (12).

A large number of biological experiments have only found a small number of genes related to esophageal cancer. In recent years, some scholars have identified esophageal cancer-related genes through computational methods such as machine learning. Liu et al. (13) identified genetic biomarkers of esophageal cancer by SALP-seq and machine learning methods. Wang et al. (14)identified the survival risk of esophageal cancer through the Kohonen network clustering algorithm and kernel extreme learning machine. Li et al. (15)used five conventional machine learning methods to identify key prognostic molecules in esophageal squamous cell carcinoma. Most of these previous studies performed gene differential expression analysis through data from a small number of patients to obtain genes related to esophageal cancer. Its sample size is insufficient and there is a sample-specific bias. It has become a trend to predict disease-related features through associations between biomolecules (16, 17). Therefore, we intend to identify esophageal cancer-related genes by their associations and correlation signatures. Through the known gene signatures associated with esophageal cancer, a computational model was constructed to explore the association of other genes with esophageal cancer.

## Materials and methods

**Figure 1:**
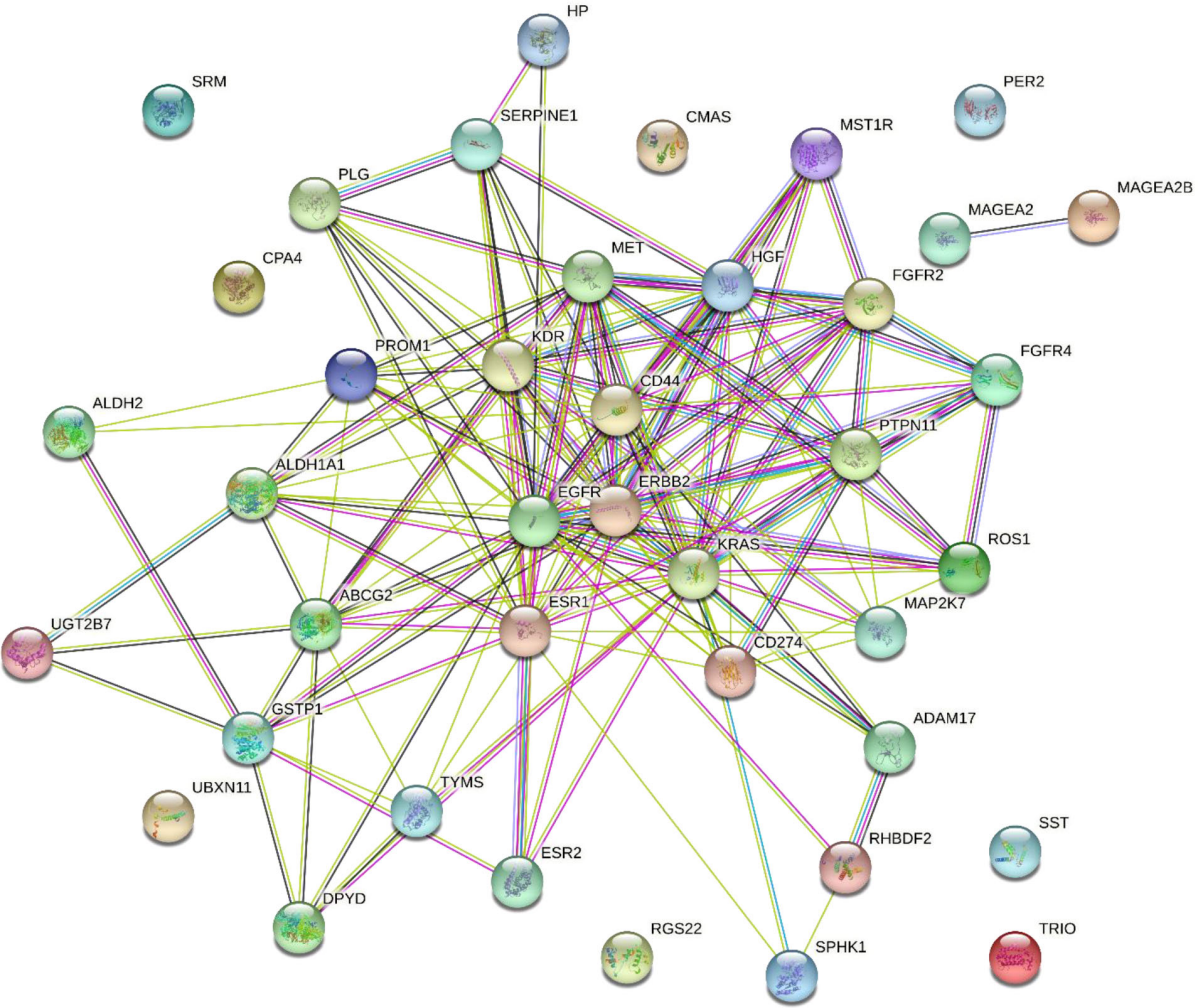
Gene interaction network of 41 esophageal cancer-related genes.

41 genes (**Supplementary Table 1**) are found to be related to esophageal cancer by DisGeNet (18). We constructed a gene interaction network by String (19), which shows as **Figure 1**.

We implemented Graph Convolutional Network (GCN) to extract feature of each gene from gene interaction network. A graph network requires the input of the node feature matrix and the adjacency matrix, so that the aggregation operation of the nodes can be performed. The input of GCN is a feature matrix A and its dimension is *N*·*F* ^0^, where N is the number of nodes in the graph network and *F* ^0^ is the number of input features per node. The adjacency matrix A matrix representation of a graph structure whose dimension is N*N.

The aggregated representation of a node does not contain its own features, the representation is the feature aggregation of neighboring nodes, so only nodes with self-loops will contain their own features in this aggregation. Therefore,


(1) $$\bar{A}=A+I$$

The propagation rules for this network are as follows:


(2) $$f(H^{i},\bar{A})=\sigma (\bar{A}H^{i}W^{i})$$

where *H^i^* is the weight matrix of the i-th layer, *σ*() is a nonlinear activation function, and the weights are shared among different nodes.

A node with a large degree will have a large value in its feature representation, and a node with a small degree will have a small value, which may cause the gradient to disappear or explode, and also affect the stochastic gradient descent algorithm. Therefore, the feature table needs to be normalized, the matrix A is multiplied by the inverse of the matrix D, and it is transformed.


(3) $$f(X,\bar{A})=D\bar{A}X$$

We implemented Logistic Matrix Factorization(LogisticMF) to identify esophageal cancer-related genes. Unlike most previous matrix factorization models, LogisticMF does not use RMSE as its loss function, but a probabilistic approach. Specifically, given an observation matrix R, it is approximated by the inner product of two low-dimensional matrices *X_mf_* an *X_mf_* , where f is the dimension of the latent factor. Definition *l_ui_* means that esophageal cancer (u) is related to gene i, and its conditional probability is given as follows:


(4) $$p(l_{ui}|x_{u},y_{i},\beta_{i},\beta_{j})=\frac{\text{exp}(x_{i}y_{i}^{T}+\beta_{u}+\beta_{i})}{1+\text{exp}(x_{i}y_{i}^{T}+\beta_{u}+\beta_{i})}$$

where *β_i_*, *β_j_* represent the bias.

Similar to Collaborative Filtering for Implicit Feedback Datasets, LogisticMF also uses confidence to represent its frequency. The confidence mapping function can take:


(5) $$c=1+a\text{log}(1+r_{ui}/\epsilon )$$

where a is a smoothing parameter that adjusts the weight of positive and negative examples.

Combining the above formula, we can get:


(6) $$L\left(R|X,Y,\beta \right)={\prod_{u,i}p\left(l_{ui}|x_{u},y_{i},\beta_{u},\beta_{j}\right)}^{ar_{ui}}\left(1-p\left(l_{ui}|x_{u},y_{i},\beta_{u},\beta_{j}\right)\right)$$

Furthermore, the underlying association matrix of esophageal cancer and genes is assumed to follow a Gaussian distribution:


(7) $$\begin{array}{c} p\left(X|\sigma^{2}\right)=\prod_{u}N\left(x_{u}|0,\sigma_{u}^{2}I\right)\\ p\left(Y|\sigma^{2}\right)=\prod_{u}N\left(y_{i}|0,\sigma_{i}^{2}I\right) \end{array}$$

Then its posterior probability is:


(8) $$\text{log }p\left(X,Y,\beta |R\right)=\sum ar_{ui}\left(x_{u}y_{i}^{T}+\beta_{u}+\beta_{i}\right)-\left(1+ar_{ui}\right)\text{log}\left(1+\text{exp}\left(x_{u}y_{i}^{T}+\beta_{u}+\beta_{i}\right)\right)-\frac{\lambda }{2}{\left|x_{u}\right|}^{2}-\frac{\lambda }{2}{\left|y_{i}\right|}^{2}$$

we should maximize the posterior probability, so use alternating gradient descent to optimize:


(9) $$\frac{\partial }{\partial x_{u}}=\sum ar_{ui}y_{i}-\frac{y_{i}\left(1+ar_{ui}\right)\text{exp}\left(x_{u}y_{i}^{T}+\beta_{u}+\beta_{i}\right)}{1+\text{exp}\left(x_{u}y_{i}^{T}+\beta_{u}+\beta_{i}\right)}-\lambda x_{u}$$

(10) $$\frac{\partial }{\partial \beta_{u}}=\sum ar_{ui}-\frac{\left(1+ar_{ui})\text{exp}(x_{u}y_{i}^{T}+\beta_{u}+\beta_{i}\right)}{1+\text{exp}(x_{u}y_{i}^{T}+\beta_{u}+\beta_{i})}-\lambda x_{u}$$

## Results

### Experiment workflow

We have obtained 41 genes which are related to esophageal cancer and wo also need negative samples to build our model. Therefore, we randomly selected 200 genes as the negative samples. We used 10-cross validation to verify the accuracy of our model. We divided our samples into 10 groups. We used nine groups of datasets to build the model and the rest one to test the model.

### Performance of GCNLMF

**Figure 2:**
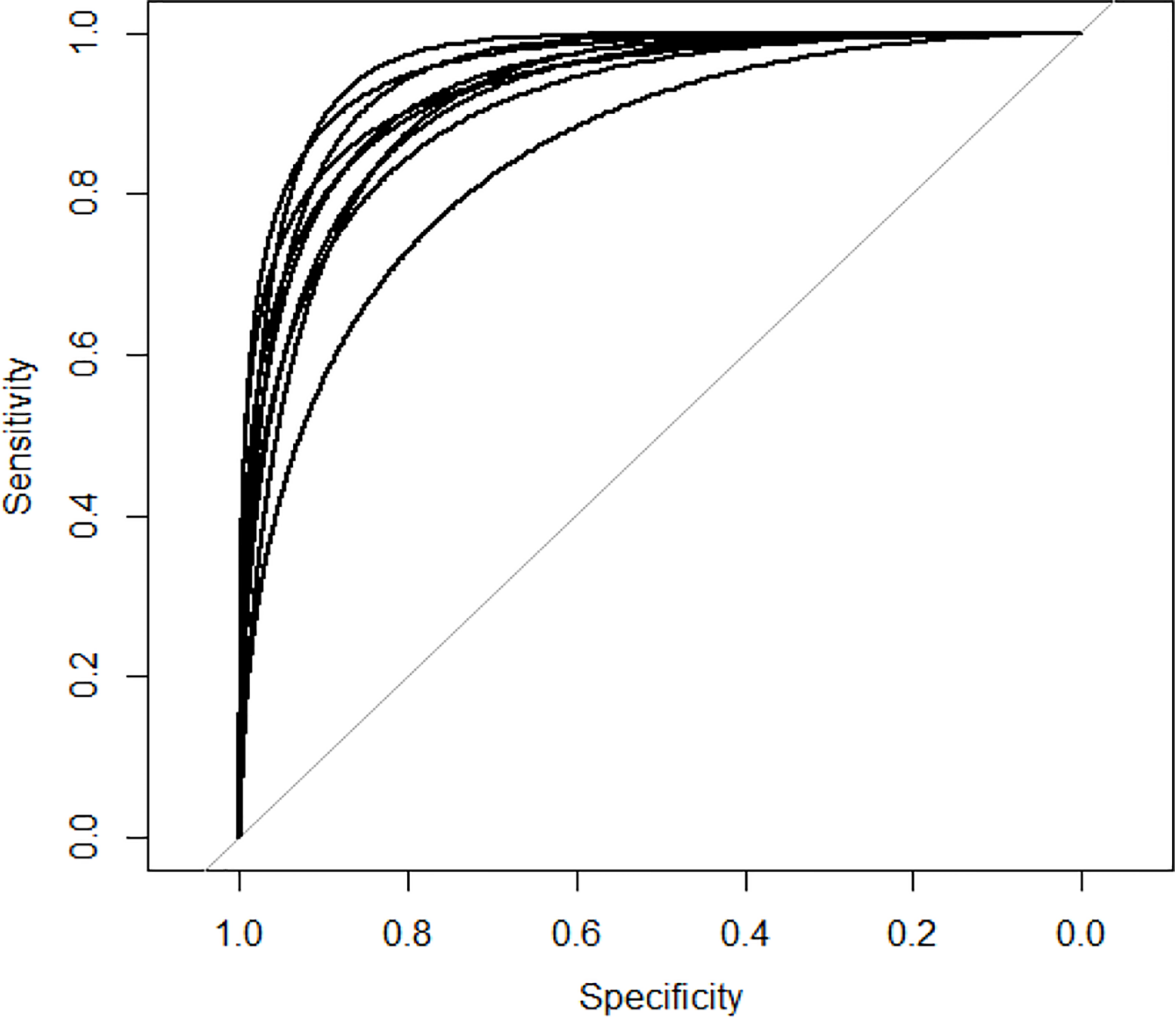
AUC curves of GCNLMF in 10-cross validation.

**Figure 3:**
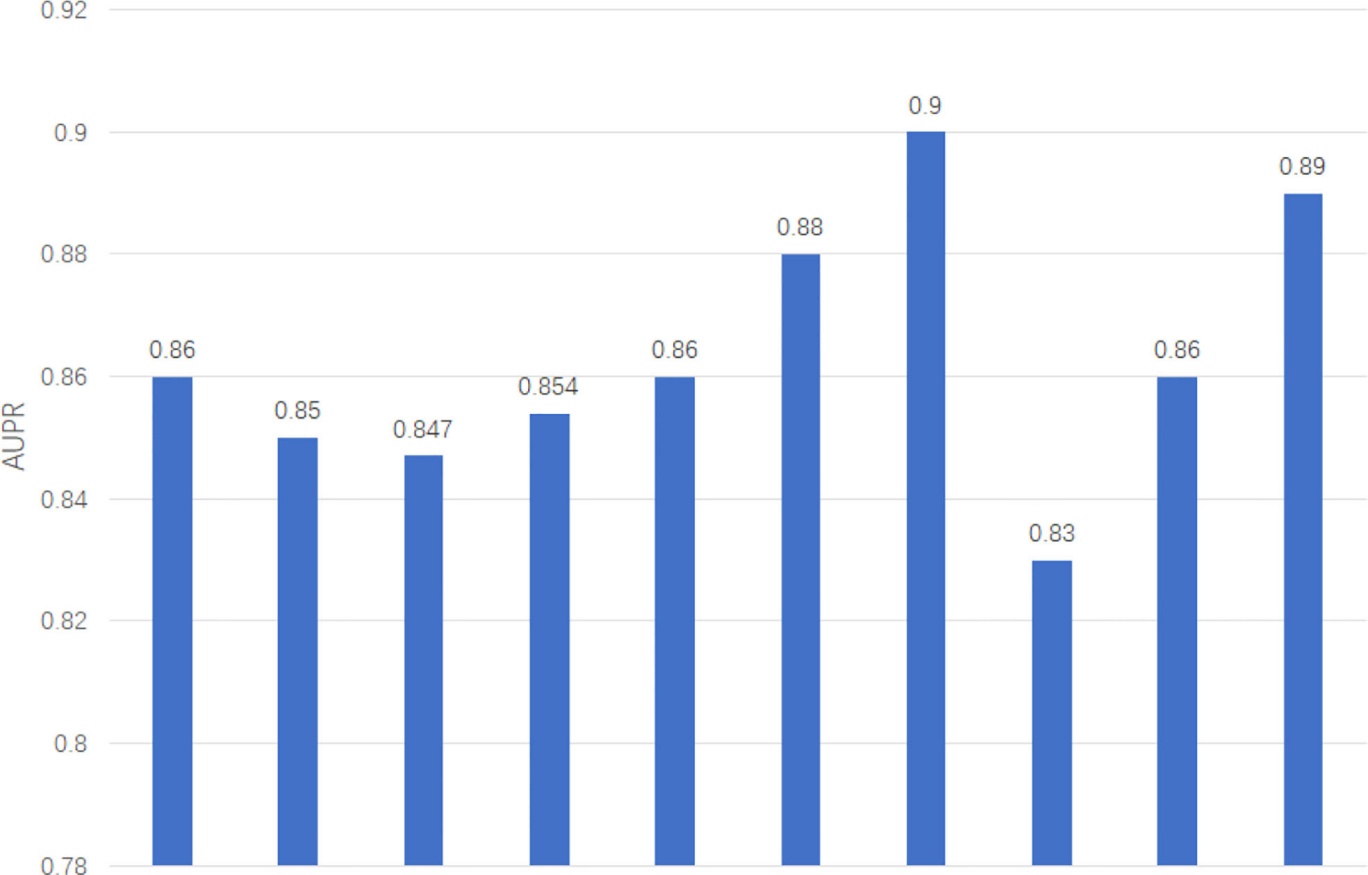
AUPR of GCNLMF in 10-cross validation.

We apply two evaluation metrics, AUC and AUPR, to evaluate our method. The experimental results of ten tests are shown in **Figures 2**, **3**.

The average of AUC is 0.927 and the standard deviation is 0.035. The average of AUPR is 0.86 and the standard deviation is 0.021. Through the cross-validation experiment, we can see that the prediction accuracy of GCNLMF is very high and stable.

### Comparison experiments

To highlight the superiority of GCNLMF, we compare it with five methods. The AUC for each method is the average value obtained by 10-fold cross-validation. The five methods include random forest (RF), gradient boosting decision tree (GBDT), GCN, LMF and Support Vector Machine(SVM). In RF, the number of decision trees was set as 100.

**Figure 4:**
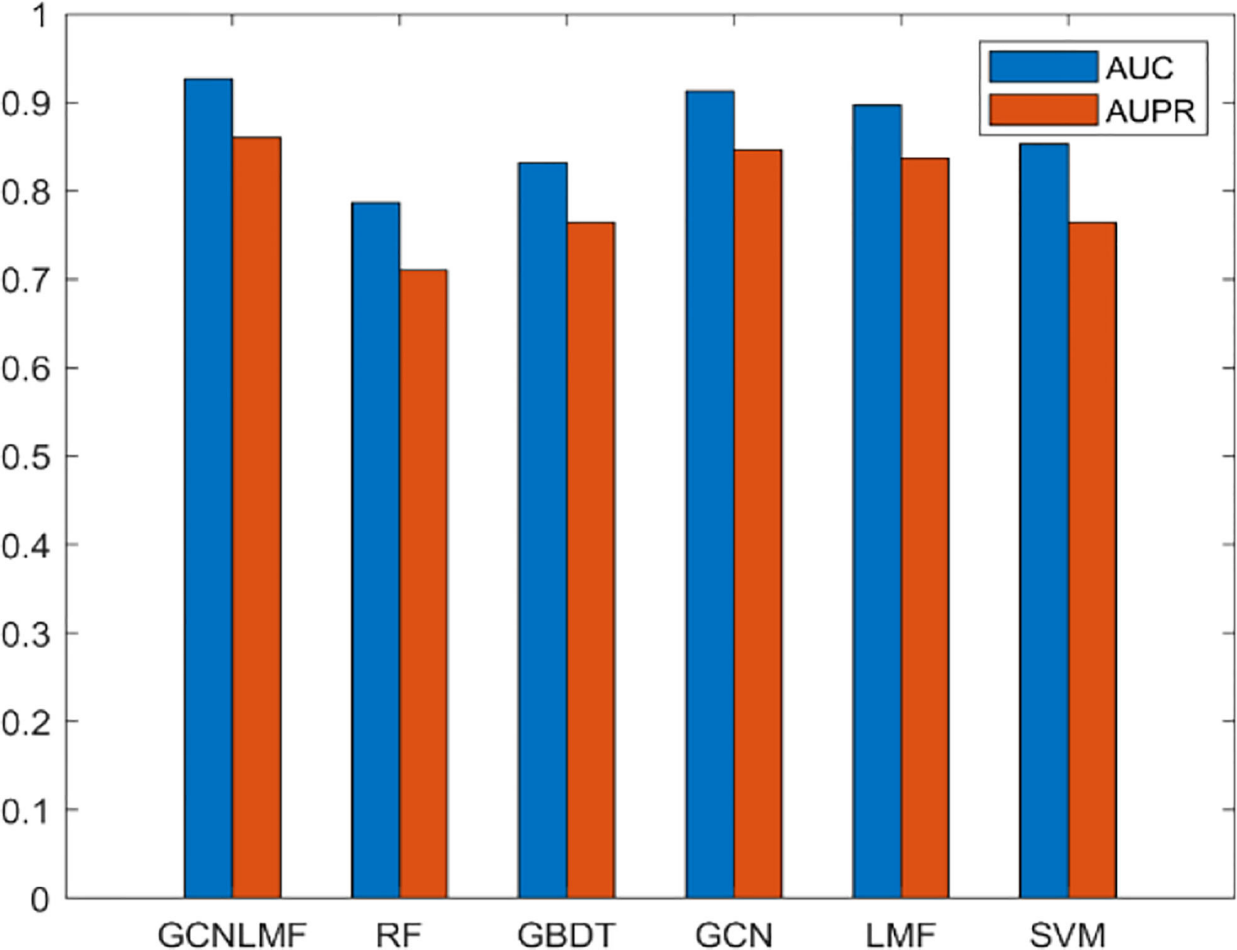
Results of GCNLMF compared to the other five methods.

The results are shown in **Figure 4**. The experiment showed that GCNLMF had the highest performance among all methods according to AUC and AUPR scores. Compared with GBDT, RF, GCN, LMF SVM, the AUC of GCNLMF increased by 14%, 9.6%, 1.4%, 3% and 7.4%, respectively. The AUPR scores increased by 15%, 9.7%, 1.4%, 2.3% and 9.6%, respectively.

### Case study

After building GCNLMF model, we used it to predict novel esophageal cancer-related genes. IL-10 is not reported to be related to esophageal cancer in the public database and GCNLMF predicted it as an esophageal cancer-related gene. Yang et al. (20) found that the -1082g/a rs1800896 genetic variation can be used as a candidate biomarker to predict the susceptibility of esophageal cancer by comparing the IL10 genotypes of 246 pathologically confirmed esophageal cancer patients and 492 healthy control subjects. Sun et al. (21) found that ETV5 was upregulated in Esophageal squamous cell carcinoma and was associated with tumor staging and prognosis. Knockdown of ETV5 or its downstream genes SKA1 and TRPV2 significantly suppress Esophageal squamous cell carcinoma cells migration and invasion, respectively. Kuerbanjiang et al. (22) detected the expression of BRAF in esophageal cancer samples by tissue microarray, and the results showed that BRAF plays an important role in the proliferation, invasion and metastasis of esophageal cancer, and overexpression of BRAF leads to shortened overall survival.

## Conclusions

The incidence of esophageal cancer has obvious familial aggregation phenomenon, which is related to the susceptibility of the population and environmental conditions. In areas with high incidence of esophageal cancer, it is not uncommon for families to have esophageal cancer patients for 3 or more consecutive generations. Therefore, it is important to discover the genetic factors of esophageal cancer.

Most previous studies have compared esophageal cancer patients with healthy people by means of DNA sequencing and RNA sequencing, so as to find gene mutations and abnormal gene expression associated with esophageal cancer. However, the time and money costs of such methods are high. At the same time, the sample size is limited and there are differences between samples. As a result, the numbers of genes associated with esophageal cancer were both small and inaccurate. Our previous studies have also confirmed the critical role of key genes and signaling pathways in the progression of esophageal cancer (23–25). This paper proposes a method GCNLMF for large-scale identification of esophageal cancer-related genes, which can effectively identify the characteristics of esophageal cancer-related genes. Through the correlation and characteristics between genes, more genes related to esophageal cancer can be predicted.

In order to verify the accuracy of GCNLMF, we used 10-cross validation. The AUC of GCNLMF was 0.927 and the aupr was 0.86 And in ten experiments, the standard deviation of these two indicators is very small, which shows that the method is robust. We also compare GCNLMF with five other commonly used methods, and we find that the accuracy of GCNLMF is significantly higher than other methods. In order to verify the accuracy of the esophageal cancer-related genes predicted by GCNLMF, we selected the top 3 genes in the prediction results to conduct a case study. Although the association of these three genes with esophageal cancer has not been reported in the database, studies by other reseachers have shown that these three genes are significantly associated with esophageal cancer, which illustrates the accuracy of the prediction results of GCNLMF.

## Data availability statement

The datasets presented in this study can be found in online repositories. The names of the repository/repositories and accession number(s) can be found in the article/**Supplementary Material**.

## Ethics statement

Ethical review and approval was not required for the study on human participants in accordance with the local legislation and institutional requirements. Written informed consent for participation was not required for this study in accordance with the national legislation and the institutional requirements.

## Author contributions

XH and FM participated in its design. W-SL, Z-GQ, LZ, H-ML, Y-WH and Y-MZ interpreted and analyzed the data. XH, W-SL and G-sZ wrote the paper. All authors contributed to the article and approved the submitted version.

## Funding

Financial support comes from the National Natural Science Foundation of China (No. 81960438, 82103067), Natural Science Foundation of Jiangxi Province (No. 20212BAB206078) and Jiangxi Provincial Education Foundation (No. GJJ190790, GJJ190792).

## Conflict of interest

The authors declare that the research was conducted in the absence of any commercial or financial relationships that could be construed as a potential conflict of interest.

## Publisher’s note

All claims expressed in this article are solely those of the authors and do not necessarily represent those of their affiliated organizations, or those of the publisher, the editors and the reviewers. Any product that may be evaluated in this article, or claim that may be made by its manufacturer, is not guaranteed or endorsed by the publisher.
